# Co-occurrence of mycotoxins and other fungal metabolites in total mixed rations of cows from dairy farms in Punjab, Pakistan

**DOI:** 10.1007/s12550-023-00502-5

**Published:** 2023-09-04

**Authors:** Felipe Penagos-Tabares, Mubarik Mahmood, Muhammad Zafar Ullah Khan, Hafiz Muhammad Amjad Talha, Muhammad Sajid, Kanwal Rafique, Saima Naveed, Johannes Faas, Juan Ignacio Artavia, Michael Sulyok, Anneliese Müller, Rudolf Krska, Qendrim Zebeli

**Affiliations:** 1 Unit of Nutritional Physiology, Institute of Physiology, Pathophysiology, and Biophysics, Department of Biomedical Sciences, University of Veterinary Medicine Vienna, Veterinärplatz 1, 1210 Vienna, Austria; 2 Department for Farm Animals and Veterinary Public Health, Christian-Doppler-Laboratory for Innovative Gut Health Concepts in Livestock (CDL-LiveGUT), University of Veterinary Medicine Vienna, Veterinärplatz 1, 1210 Vienna, Austria; 3 FFoQSI GmbH – Austrian Competence Centre for Feed and Food Quality, Safety and Innovation, Technopark 1C, 3430 Tulln, Austria; 4 Department of Animal Sciences, University of Veterinary and Animal Sciences, Lahore, Subcampus Jhang, 12 km Chiniot Road, Jhang, 35200 Pakistan; 5 Agri-Food Research & Sustainable Solutions (ARASS), Private Limited F-1, IBL Market, Ghouri Block, Bahria Town, Lahore, 54000 Pakistan; 6 Department of Animal Nutrition, Ravi Campus, Pattoki, University of Veterinary and Animal Sciences, Lahore, 55300 Pakistan; 7 DSM-BIOMIN Research Center, Technopark 1, 3430 Tulln an der Donau, Austria; 8 Department of Agrobiotechnology, IFA-Tulln, Institute of Bioanalytics and Agro-Metabolomics, University of Natural Resources and Life Sciences, Vienna, Konrad-Lorenz-Strasse 20, 3430 Tulln an der Donau, Austria; 9 Institute for Global Food Security, School of Biological Sciences, Queen’s University Belfast, University Road, Belfast, BT7 1NN UK; 10 Institute of Animal Nutrition and Functional Plant Compounds, Department for Farm Animals and Veterinary Public Health, University of Veterinary Medicine Vienna, Veterinärplatz 1, 1210 Vienna, Austria

**Keywords:** Feed safety, Multi-mycotoxin analysis, Dairy farm, Total mixed ration, Dairy cow

## Abstract

After India and the USA, Pakistan is the third country leading in global dairy production, a sector of very high socioeconomic relevance in Asia. Mycotoxins can affect animal health, reproduction and productivity. This study analysed a broad range of co-occurring mycotoxins and fungal secondary metabolites derived from *Alternaria*, *Aspergillus*, *Fusarium*, *Penicillium* and other fungal species. To complete this, a validated multi-metabolite liquid chromatography/electrospray ionization-tandem mass spectrometric (LC/ESI–MS/MS) method was employed, detecting 96 of > 500 tested secondary fungal metabolites. This first preliminary study demonstrated that total mixed rations (TMRs) (*n* = 30) from big commercial dairy cattle farms (> 200 lactating cows) in Punjab, Pakistan, presented ubiquitous contamination with mixtures of mycotoxins. The mean of mycotoxins per sample was 14, ranging from 11 to 20 mycotoxins among all TMR samples. Metabolites derived from other fungi and *Fusarium* spp. showed the highest levels, frequency and diversity among the detected fungal compounds. Among the most prevalent mycotoxins were *Fusarium* toxins like fumonisins B1 (FB1) (93%), B2 (FB2) (100%) and B3 (FB3) (77%) and others. Aflatoxin B1 (AFB1) was evidenced in 40% of the samples, and 7% exceeded the EU maximum limit for feeding dairy cattle (5 µg/kg at 88% dry matter). No other mycotoxin exceeds the EU guidance values (GVs). Additionally, we found that dietary ingredients like corn grain, soybean meal and canola meal were related to increased contamination of some mycotoxins (like FB1, FB2 and FB3) in TMR from the province of Punjab, Pakistan. Among typical forage sources, the content of maize silage was ubiquitous. Individually, the detected mycotoxins represented relatively low levels. However, under a realistic scenario, long-term exposure to multiple mycotoxins and other fungal secondary metabolites can exert unpredictable effects on animal health, reproduction and productivity. Except for ergot alkaloids (73%), all the groups of metabolites (i.e. derived from *Alternaria* spp., *Aspergillus* spp., *Fusarium* spp., *Penicillium* spp. and other fungi) occurred in 100% of the TMR samples. At individual levels, no other mycotoxins than AFB1 represented a considerable risk; however, the high levels of co-occurrence with several mycotoxins/metabolites suggest that long-term exposure should be considered because of their potential toxicological interactions (additive or synergistic effects).

**Supplementary Information:**

The online version contains supplementary material available at 10.1007/s12550-023-00502-5.

## Introduction

Located in Asia, the continent with the highest milk production worldwide, Pakistan is the third major milk producer after India and the USA (Food and Agriculture Organization of the United Nations 2021). The dairy cow diets contain various ingredients, including roughages, cereal grains and agro-industrial by-products (FAO, IDF, IFCN 2014). Crops and feedstuffs are vulnerable to mould infection and colonization with successive mycotoxin contamination during the complete feed production chain (pre- and post-harvest) influenced by several biotic and abiotic factors. The livestock industry endures severe economic losses due to the adverse effects of contaminated feed on animal health and the final quality of the products (Bryden 2012). The climatic conditions of Pakistan typically favour mycotoxin contamination in agricultural commodities (Ashiq 2015). According to a survey, South Asia was, in the last decade, the world’s region with the highest occurrence of aflatoxin B1 (AFB1) (82%) in feed samples. South Asia, along with Sub-Saharan Africa, showed the highest median values of AFB1-positive feed samples (≥ 20 µg/kg) (Gruber-Dorninger et al. 2019). AFB1 is a public health concern because of its proven carcinogenic properties (Massey et al. 1995). Previous studies on aflatoxin M1 (AFM1) in the Punjab Province of Pakistan indicated that 99%, 32% and 58% of the milk samples evaluated in the respective studies exceeded the European Union and Codex Alimentarius limit (0.05 µg/L), which indicates the constant exposure of dairy products’ consumers to aflatoxins (Codex Alimentarius Commission 2001; Hussain and Anwar 2008; Iqbal and Asi 2013; Sadia et al. 2012). As feed is the central source of AFM1 in cow milk, the level of aflatoxins in dairy cattle diets should also be monitored and kept to a minimum (Sadia et al. 2012).

Previous surveillance studies on contamination of dairy cattle feed in Pakistan focused mainly on aflatoxins (AFs), zearalenone (ZEN), ochratoxin A (OTA) and trichothecenes (types A and B) (Ashiq 2015; Aslam and Wynn 2015; Gallo et al. 2015; Gruber-Dorninger et al. 2019; Santos Pereira et al. 2019; Yunus et al. 2020; Akbar et al. 2020). The most relevant investigated mycotoxins include the strictly regulated AFB1 and other mycotoxins with GV addressed by the EU legislation like deoxynivalenol (DON), ZEN, fumonisins (FBs), OTA as well as T-2 and HT-2 toxins (EC 2002, 2006; Gallo et al. 2015; Gruber-Dorninger et al. 2019). Although hundreds of compounds have been considered mycotoxins, most of the relevant studies investigated a limited number of mycotoxins in agricultural commodities (Gallo et al. 2015; Cinar and Onbaşı 2019; Battilani et al. 2020). Toxicological interactions (addition, synergism, potentiation and antagonism) among mycotoxins and other fungal metabolites affect animal and human health and reproduction (Smith et al. 2016). This requires more research and risk assessment by more integrative approaches (Battilani et al. 2020). Multi-mycotoxin contamination has been evidenced at pre-harvest and post-harvest (Rasmussen et al. 2010; Nichea et al. 2015a, b; Panasiuk et al. 2019; Hajnal et al. 2020; Penagos-Tabares et al. 2021, 2022a). It has been evidenced that dairy cattle diets such as total mixed rations (TMRs) are generally contaminated with complex cocktails of dozens of mycotoxins and other fungal and plant metabolites (Awapak et al. 2021; Penagos-Tabares et al. 2022b). TMR is a “complete ration” feeding system, which is very popular worldwide on dairy farms with big herds. TMR is produced by mixing forages, by-products, cereal grains, concentrates, minerals, vitamins and additives. From this mix, animals get the nutrients needed to meet maintenance and production requirements (Bueno et al. 2020; Schingoethe 2017).

Sub-clinical disorders in dairy cows, such as disrupted rumen function or increased susceptibility to infections, might be related to the impact of complex mixtures of toxic fungal secondary metabolites (Santos and Fink-Gremmels 2014). The relevance of synergistic interactions and consequences of long-term exposure to such mycotoxin mixtures is recognized, and the importance of integrative and innovative approaches based on multi-mycotoxin analyses has been highlighted (Battilani et al. 2020). Therefore, this investigation planned to determine the frequency, co-occurrences and concentration of contamination with mycotoxins and other fungal metabolites (> 500) derived from species of *Alternaria*, *Aspergillus*, *Fusarium*, *Penicillium* and other fungi in the TMR samples of dairy cattle farms from Punjab, Pakistan. The analysis was accomplished by employing a validated multi-metabolite liquid chromatography/electrospray ionization-tandem mass spectrometric (LC/ESI–MS/MS) method. The possible relationship of the main dietary ingredients to the dietary concentrations of mycotoxins and other metabolites was also explored.

## Materials and methods

### Sampling and sample preparation

**Fig. 1:**
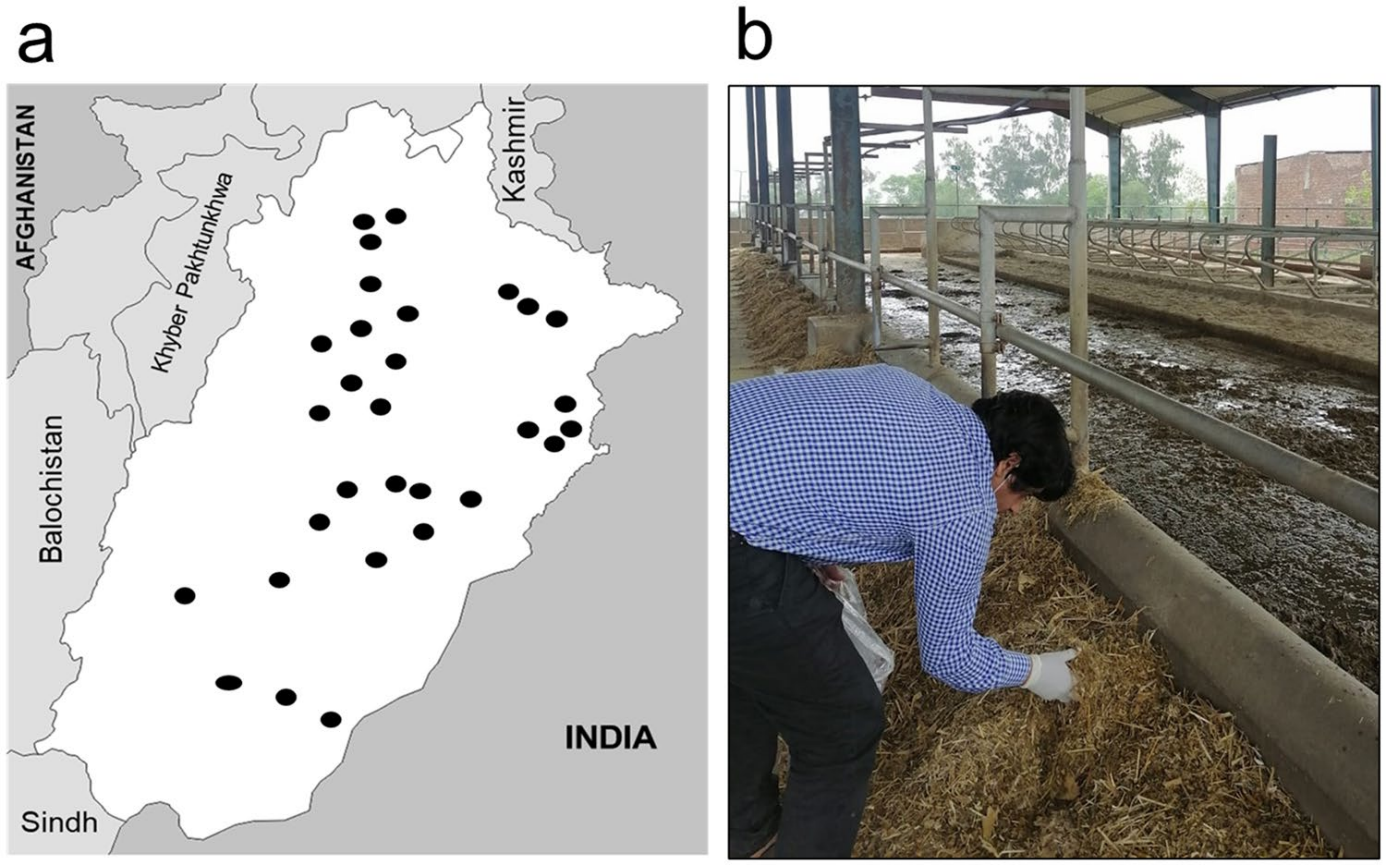
Representative sampling of total mixed rations (TMRs) from dairy farms in Punjab, Pakistan. **a** Map of the province of Punjab, illustrating the localization of explored farms. **b** The representative sampling consisted of at least 30 incremental (handful) samples collected from the feeding table immediately after serving

After obtainment of written authorization and consent of the farmers, TMR (*n* = 30) samples were collected from corporate dairy farms in Punjab, Pakistan (Fig. 1a). The herd size of the farms was over 200 Holstein-Frisian lactating cows. The farms were selected so that each of the nine administrative divisions of Punjab contributed at least three farms. Information regarding the TMR composition (main ingredients, proportions and estimated feed intake) was provided by the farmers (*n* = 29/30) via a personal (questionnaire-guided) interview. Each representative sample of TMR consisted of a minimum of 30 incremental samples, which were manually collected from the feed bunk with gloves directly after the serving (Fig. 1b). The final TMR sample amount was 1–1.5 kg, which was mixed and immediately vacuum-packed and stored in the dark at − 20 °C. Sampling was carried out during the period June–July of 2020. For the sample preparation, the frozen TMRs were thawed at room temperature for 24 h and air-dried at 65 °C for 48 h. Then, the dried TMRs were milled to a final particle size of ≤ 0.5 mm, using the cutting mill (SM 300; Retsch GmbH, Haan, Germany) at 1500 rpm for approximately 1 min, and the remnants (> 0.5 mm) were processed using an ultra-centrifugal mill (ZM 200; Retsch GmbH, Haan, Germany) at 10,000 rpm for approximately 30 s, following the procedures described by Penagos-Tabares et al. (2022a, b). Finally, 5 g (± 0.01 g) of each homogenized TMR sample was weighed into 50-mL polypropylene conical tubes (Sarstedt, Nümbrecht, Germany) and stored at − 20 °C until posterior analysis targeting multiple mycotoxins and other fungal secondary metabolites.

### Multi-mycotoxin analysis (LC/ESI–MS/MS)

Water purification was done using a Purelab Ultra system (ELGA LabWater, Celle, Germany). Glacial acetic acid (p.a.) and ammonium acetate (LC-MS grade) were purchased from Sigma-Aldrich (Vienna, Austria). HiPerSolv Chromanorm HPLC gradient grade acetonitrile was obtained from VWR Chemicals (Vienna, Austria), and LC-MS Chromasolv grade methanol was acquired from Honeywell (Seelze, Germany). Standards of fungal, plant and unspecific secondary metabolites were purchased from several commercial suppliers or obtained via a donation from different research institutions (Sulyok et al. 2020). For simultaneous multiple metabolite quantification, 5 g (± 0.01 g) of the TMR sample was extracted in 20 mL of the extraction solvent (acetonitrile/water/acetic acid 79:20:1, v/v/v) following the procedures reported by Sulyok et al. (2020). These volumes were put into the QTrap 5500 LC-MS/MS system (Applied Biosystems, Foster City, CA, USA) equipped with a Turbo V electrospray ionization (ESI) source coupled to a 1290 series UHPLC system (Agilent Technologies, Waldbronn, Germany, as described by Sulyok et al. 2020). Subsequently, quantification from external calibration by serial dilutions of a stock solution of analysed compounds was accomplished. In the end, the results were adjusted for apparent recoveries defined through spiking experiments according to Steiner et al. (2020). This analytical methodology has been validated (Steiner et al. 2020; Sulyok et al. 2020) and has been utilized to study the multi-mycotoxin occurrence in complex feedstuff matrices like silage, pastures, concentrate feed and TMR (Shimshoni et al. 2013; Nichea et al. 2015b; Reisinger et al. 2019; Awapak et al. 2021; Penagos-Tabares et al. 2021, 2022a, b). The method accuracy has been verified on a routine basis by proficiency testing organized by BIPEA (Gennevilliers, France). Satisfactory *z*-scores between − 2 and 2 have been obtained for > 95% of > 1700 results submitted so far.

### Statistical analysis

Concentrations of mycotoxins and other fungal metabolites were presented on a dry matter basis in µg/kg. Descriptive statistics (i.e. occurrences, mean, median and range of the concentrations of mycotoxins and metabolites) were processed, considering only the positive values (*x* ≥ limit of detection (LOD) using Microsoft^®^ Excel^®^. Values between the limit of quantification (LOQ) and LOD were calculated as LOQ/2. A two-tailed Spearman’s correlation test was conducted to explore possible relationships between dietary compounds and levels of metabolites, as well as relationships among metabolites within each ingredient compound. For this, only data of metabolites with frequencies over 30% was studied. Spearman’s correlation coefficients were considered significant at a *p* value < 0.05. Accordingly, the correlation coefficients were interpreted according to Hinkle et al. (2003) as follows: “very high” (0.90 up to 1.00), “high” (0.70 up to 0.90), “moderate” (0.50 up to 0.70), “low” (0.30 up to 0.50) and “negligible” (< 0.30). Low and negligible correlations were not considered during interpretation in the results’ description. Linear regressions between fungal metabolites and the content of certain feed ingredients were performed to corroborate the promising relationships. The statistical analyses and graphs were performed using GraphPad Prism version 9.1 (GraphPad Software, San Diego, CA, USA).

## Results

### Main dietary components

The selected farms fed TMR, which is a feeding method consisting of complete diet composed of the mixtures of forages and varying quantities of concentrate feed, by-products and mineral supplements. The frequency and rate of the inclusion levels of the main TMR ingredients offered to lactating cows for all selected farms are shown in Table 1. The most common dietary components included were maize silage (100%), commercial concentrate (90%), corn (maize) grain (83%), soybean meal (83%), canola meal (79%), molasses (72%), wheat straw (52%), Rhodes grass hay (34%), rice polish (21%) and wheat bran (21%). Other feedstuffs, including lucerne hay, rapeseed cake, palm kernel cake, maize gluten, lucerne, sugar beet pulp, cotton seed cake and rice bran, were less frequently (< 20%) included (Table 1). Proportionally, maize silage was the most abundant dietary ingredient, with an average inclusion of 65.1% DM of the ration, varying from 41.6 to 77%. Maize grain was incorporated on an average proportion of 10.2% of the ration (DM basis), followed by commercial concentrate (8.7%), lucerne hay (6.1%) and soybean meal (6.1%). On average, the TMR samples contained 30.4% of concentrate feeds and 69.6% of forages. The forage-to-concentrate ratio (F:C) fluctuated between 52:48 and 84:16 (Table 1).

**Table 1:** Frequencies and proportion of inclusion of the main components incorporated in total mixed rations (*n* = 29) of dairy farms in Punjab, Pakistan

**Dietary ingredient**	**Frequency of inclusion**^a^, ***n*** **(%)**	**Proportion of inclusion (% DM)**		
		**Average ± SD**	**Median**	**Range**
**Maize silage**	29 (100)	65.1 ± 8.3	68	41.6–77
**Commercial concentrate**	26 (90)	8.7 ± 11.8	2.53	1–40
**Maize (grain)**	24 (83)	10.2 ± 3.9	10	3–19.2
**Soybean meal**	24 (83)	6.1 ± 2.3	6.1	2–12
**Canola meal**	23 (79)	5.7 ± 2.8	5	2–12
**Molasses**	21 (72)	2.5 ± 0.9	2	0.5–4
**Wheat straw**	15 (52)	3 ± 1.5	3	0.5–5
**Rhodes grass hay**	10 (34)	2.8 ± 2.1	2.5	1–8
**Rice polish**	6 (21)	2.9 ± 1.1	2.5	2–4.4
**Wheat bran**	6 (21)	3.1 ± 2	2.6	0.6–5.8
**Lucerne hay**	4 (14)	6.1 ± 3.1	5.5	3–10.4
**Rapeseed cake**	4 (14)	2.5 ± 1.3	2.5	1.1–4
**Palm kernel cake**	2 (7)	4 ± 0	4	4–4
**Maize gluten**	2 (7)	2.5 ± 0.7	2.5	2–3
**Lucerne**	1 (3)			35
**Sugar beet pulp**	1 (3)			15
**Cotton seed cake**	1 (3)			1
**Rice bran**	1 (3)			2
**Peanut kari**	1 (3)			2
**Black lentils**	1 (3)			1
**Concentrate %**	29 (100)	30.4 ± 7.2	28	16–48
**Forage %**	29 (100)	69.6 ± 7.2	72	52–84

^a^*n* = 29, one sampled farm declined to provide the information on the total mixed ration (TMR) composition

### Occurrence and concentrations of mycotoxins and other secondary metabolites

#### General overview

**Fig. 2:**
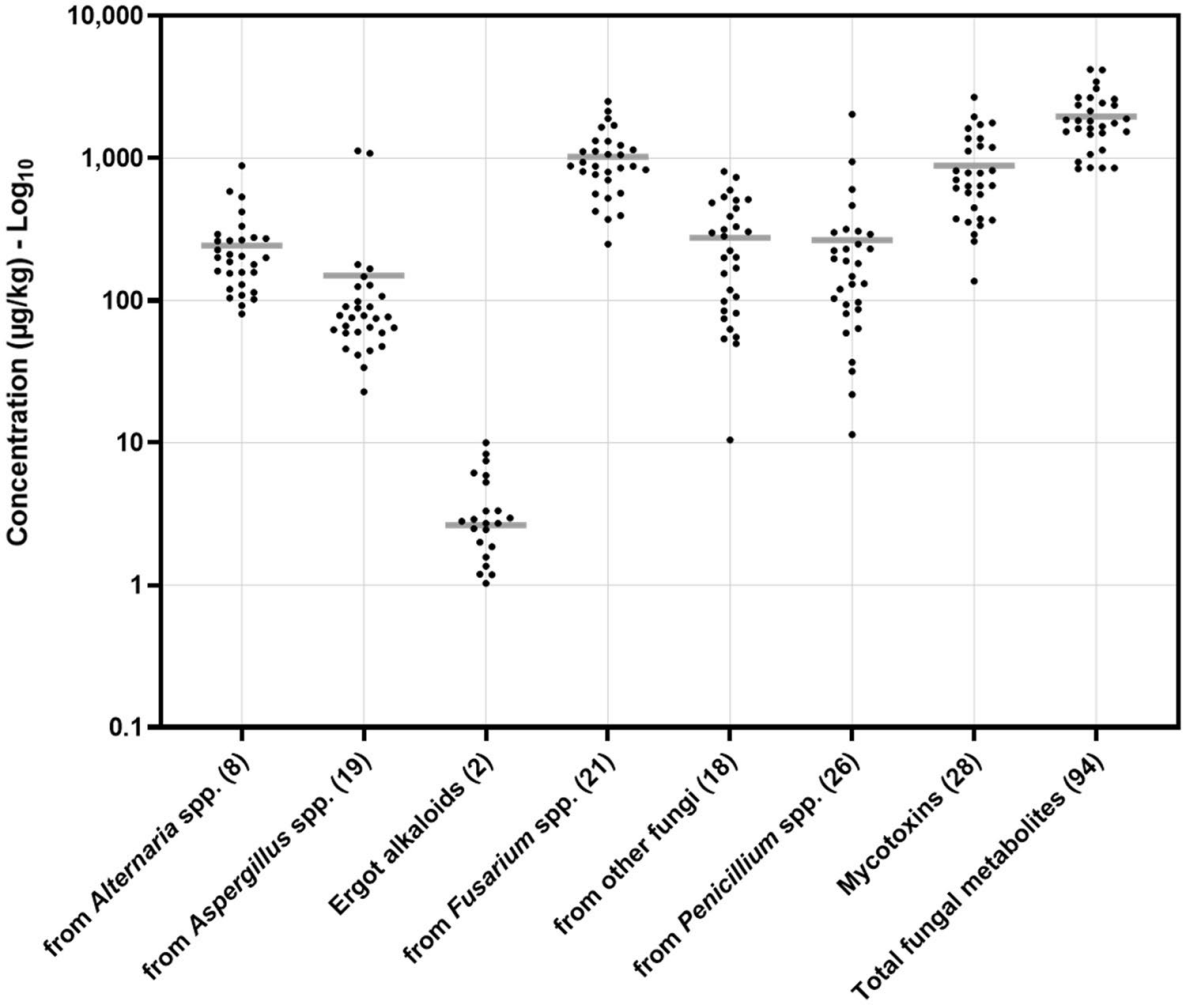
Distribution of the concentration of categorized mycotoxins and other fungal metabolites detected in the TMR samples in Punjab, Pakistan. The total number of metabolites detected per group is shown in parentheses

This study identified 96 mycotoxins and fungal secondary metabolites that contaminated TMR intended for feeding dairy cows in Pakistan. The analytes were classified by their main producers based on previous reports (Szulc et al. 2019; Hajnal et al. 2020; Penagos-Tabares et al. 2021, 2022a, b). Metabolites of *Penicillium* spp. (27), *Fusarium* spp. (21), other fungi (19), *Aspergillus* spp. (19), *Alternaria* spp. (8) and ergot alkaloids (EAs) (2) were detected. Except for ergot alkaloids (73%), all the mentioned categories were found in 100% of the samples. Figure 2 illustrates the mentioned groups’ occurrences and concentrations (mean, maximum and minimum). The metabolites produced by *Fusarium* spp. showed the highest concentrations (average ± SD: 1020 µg/kg ± 531 µg/kg, range: 249–2510 µg/kg), followed by the groups of analytes from other fungal species (276 µg/kg ± 217 µg/kg, 10.5–804 µg/kg), *Penicillium* spp. (266 µg/kg ± 386 µg/kg, 11.4–2036 µg/kg), *Alternaria* spp. (243 µg/kg ± 172 µg/kg, 80.6–887 µg/kg), *Aspergillus* spp. (149 µg/kg ± 262 µg/kg, 22.8–1125 µg/kg) and ergot alkaloids (3.59 µg/kg ± 2.51 µg/kg, 1.03–10.0 µg/kg). The accumulated concentration of fungal secondary metabolites was, on average, 1960 µg/kg ± 909 µg/kg, fluctuating from 842 to 4196 µg/kg (Fig. 2).

### Occurrence of individual mycotoxins and other secondary fungal metabolites

Concerning mycotoxins contemplated in international legislation, AFB1 was detected in 40% of the samples ranging from 1.10 to 33.8 µg/kg. Seven percent of the samples exceeded the maximum levels of AFB1 allowed by EU legislation (5 µg/kg on 88% DM). The sample with the highest AFB1 values (33.8 µg/kg) was also the only sample co-contaminated with AFB2 (6.51 µg/kg) and AFM1 (1.18 µg/kg). The occurrences and levels (mean, median and range) and the mycotoxin/metabolite levels are presented in Table 2. Three fumonisins were highly occurrent: FB1 (93%), FB2 (100%) and FB3 (77%); the detected levels (maximum of FB1 + FB2: 383 µg/kg) are below the GV of the EU for the sum of FB1 and FB2 for complementary and complete feeding stuffs for dairy cattle (50,000 µg/kg) (EC 2006). ZEN occurred in 43% of the TMR samples, on average 13.1 µg/kg, ranging from 2.94 to 57.2 µg/kg. OTA was detected in 7% of the samples in a concentration below 35 µg/kg. No sample exceeded the EU GVs of ZEN (500 µg/kg), the sum of FB1 and FB2 (50,000 µg/kg) and OTA (250 µg/kg) for complementary and complete feeding stuffs for dairy cattle (EC 2006). All samples were negative for DON, HT-2 toxin and T-2 toxin. Nivalenol (NIV) was detected in 40% of the samples, ranging from 121 to 1310 µg/kg. Chrysogin, culmorin, deoxyfusapyron, enniatins B and B1, fusaproliferin, fusapyron and gibberellin A12 occurred at ≤ 20% and below 40 µg/kg (Table 2). Emerging mycotoxins, like beauvericin, bikaverin, epiequisetin and equisetin, were found in 100% of the samples. Monocerin (93%) and moniliformin (87%) presented the highest occurrences with concentrations lower than 105 µg/kg.

**Table 2:** Occurrences and levels of mycotoxins and other fungal metabolites detected in total mixed rations of dairy farms in Punjab, Pakistan

**Group**	**Metabolites**	**Occurrence**^a^, ***n*** **(%)**	**Concentration (µg/kg)**^b^		
			**Average ± SD**	**Median**	**Range**
***Alternaria* spp.**	**Altenuisol**	2 (7)	14.9 ± 2.61	14.9	13.1–16.8
	**Alternariol**	12 (40)	2.71 ± 3.08	1.38	1.08–11.3
	**Alternariolmethylether**	27 (90)	3.09 ± 3.16	2.30	1.04–17.2
	**Altertoxin I**	1 (3)			3.62
	**Infectopyron**	27 (90)	94.8 ± 107	54.3	5.87–400
	**Macrosporin**	3 (10)	1.63 ± 0.69	1.36	1.12–2.42
	**Tentoxin**	30 (100)	9.24 ± 4.52	7.83	2.84–20.1
	**Tenuazonic acid**	29 (97)	148 ± 87.3	118	71.8–492
***Aspergillus* spp.**	**Aflatoxin B1**	12 (40)	6.19 ± 9.26	2.39	1.10–33.8
	**Aflatoxin B2**	1 (3)			6.51
	**Aflatoxin M1**	1 (3)			1.18
	**Averufin**	21 (70)	2.35 ± 1.61	1.78	1.00–6.23
	**Bis(methylthio)gliotoxin**	5 (17)	7.32 ± 6.99	3.56	2.02–19.0
	**Deoxygerfelin**	2 (7)	1.38 ± 0.41	1.38	1.09–1.67
	**Integracin A**	1 (3)			22.1
	**Integracin B**	1 (3)			11.5
	**Kojic acid**	30 (100)	134 ± 245	61.5	22.8–1060
	**Kotanin A**	1 (3)			2.32
	**Malformin A**	1 (3)			32.1
	**Malformin C**	1 (3)			4.07
	**Norsolorinic acid**	3 (10)	11.6 ± 13.6	5.50	2.11–27.2
	***O*-Methylsterigmatocystin**	1 (3)			1.50
	**Pinselin**	8 (27)	6.10 ± 4.41	4.27	1.59–12.8
	**Seco-sterigmatocystin**	8 (27)	2.61 ± 1.19	2.29	1.40–5.05
	**Sterigmatocystin**	11 (37)	4.07 ± 3.06	3.64	1.13–9.99
	**Sydonol**	2 (7)	7.04 ± 0.91	7.04	6.40–7.68
	**Versicolorin C**	19 (63)	2.31 ± 1.47	1.89	1.01–6.54
**Ergot alkaloids**	**Ergometrinine**	22 (73)	3.52 ± 2.46	2.76	1.03–10.0
	**Ergosinine**	1 (3)			1.64
***Fusarium* spp.**	**Beauvericin**	30 (100)	21.1 ± 19.4	17.0	2.71–107
	**Bikaverin**	30 (100)	28.0 ± 28.2	21.5	2.03–150
	**Chrysogin**	5 (17)	22.4 ± 11.07	24.9	6.92–35.3
	**Culmorin**	3 (10)	31.4 ± 8.30	35.2	21.9–37.1
	**Deoxyfusapyron**	1 (3)			9.13
	**Enniatin B**	6 (20)	2.13 ± 1.45	1.47	1.16–4.91
	**Enniatin B1**	2 (7)	1.48 ± 0.54	1.48	1.10–1.86
	**Epiequisetin**	30 (100)	6.90 ± 4.07	6.40	1.07–14.7
	**Equisetin**	30 (100)	31.6 ± 20.7	27.9	4.25–83.1
	**Fumonisin A1 precursor**	2 (7)	6.72 ± 3.35	6.72	4.35–9.09
	**Fumonisin B1**	28 (93)	111 ± 67.0	98.7	25.4–274
	**Fumonisin B2**	30 (100)	45.2 ± 27.4	42.2	7.30–109
	**Fumonisin B3**	23 (77)	27.8 ± 12.2	22.9	11.9–54.8
	**Fusaproliferin**	1 (3)			39.0
	**Fusapyron**	3 (10)	1.80 ± 0.41	1.97	1.33–2.09
	**Gibberellin A12**	5 (17)	29.4 ± 21.1	23.10	11.7–65.5
	**Moniliformin**	26 (87)	13.6 ± 14.5	9.47	3.96–76.8
	**Monocerin**	28 (93)	19.8 ± 28.5	10.1	2.05–104
	**Nivalenol**	12 (40)	475 ± 403	284	121–1310
	**Siccanol**	29 (97)	542 ± 404	396	144–1900
	**Zearalenone**	13 (43)	13.1 ± 17.8	5.97	2.94–57.2
**Other fungi**	**Ascochlorin**	29 (97)	9.16 ± 6.48	8.58	1.21–28
	**Barceloneic acid**	13 (43)	191 ± 163	117	52.8–588
	**Bassianolide**	2 (7)	1.80 ± 0.04	1.80	1.77–1.83
	**Cercosporin**	8 (27)	28.0 ± 27.3	19.9	3.92–74
	**Cladosporin**	1 (3)			19.9
	**Clonostachydiol**	3 (10)	3.64 ± 1.88	2.75	2.38–5.80
	**Cytochalasin B**	6 (20)	46.0 ± 38.3	38.6	11.6–115
	**Cytochalasin D**	26 (87)	20.4 ± 16.5	15.9	1.17–80.6
	**Cytochalasin J**	4 (13)	30.3 ± 24.1	20.1	14.9–66.3
	**Destruxin B**	23 (77)	18.6 ± 40.4	6.52	1.16–199
	**Ilicicolin A**	2 (7)	1.43 ± 0.51	1.43	1.07–1.79
	**Ilicicolin B**	29 (97)	34.2 ± 68.5	4.64	1.08–240
	**Ilicicolin E**	6 (20)	2.59 ± 1.99	1.89	1.01–6.35
	**LL-Z 1640-4**	3 (10)	3.41 ± 1.47	2.91	2.25–5.07
	**MER-NF5003E**	1 (3)			1.29
	**Mollicellin D**	7 (23)	13.5 ± 9.36	15.4	1.81–23.7
	**Neoechinulin A**	29 (97)	96.1 ± 145	35.6	2.93–602
	**PF 1163A**	4 (13)	3.23 ± 1.95	3.09	1.11–5.62
***Penicillium* spp.**	**7-Hydroxypestalotin**	1 (3)			9.22
	**Andrastin A**	2 (7)	4.98 ± 4.67	4.98	1.67–8.28
	**Atpenin A5**	6 (20)	3.01 ± 1.34	2.90	1.16–5.28
	**Citreohybridinol**	3 (10)	1.92 ± 0.19	1.92	1.73–2.10
	**Citreoviridin**	4 (13)	53.1 ± 87.1	11.7	5.50–184
	**Curvularin**	7 (23)	4.90 ± 3.29	3.42	2.14–10.6
	**Cycloaspeptide A**	10 (33)	26.06 ± 24.4	24.9	1.03–80.7
	**Cyclopenin**	2 (7)	1.48 ± 0.37	1.48	1.21–1.74
	**Cyclopenol**	2 (7)	15.3 ± 2.32	15.3	13.7–17.0
	**Dechlorogriseofulvin**	2 (7)	1.84 ± 0.66	1.84	1.37–2.30
	**Dehydrocurvularin**	1 (3)			9.80
	**Dihydrocitrinone**	1 (3)			37.4
	**Flavoglaucin**	30 (100)	166 ± 378	35.3	3.42–1950
	**Griseofulvin**	22 (73)	6.53 ± 6.50	4.25	1.05–24.1
	**Mycophenolic acid**	8 (27)	27.8 ± 25.3	17.07	1.07–65.6
	**Mycophenolic acid IV**	2 (7)	12.0 ± 3.64	12.0	9.39–14.5
	**Ochratoxin A**	2 (7)	17.1 ± 21.60	17.1	1.85–32.4
	**Ochratoxin B**	1 (3)			2.29
	**Oxaline**	25 (83)	39.4 ± 53.5	18.7	1.31–219
	**Penicolinate**	2 (7)	1.48 ± 0.33	1.48	1.25–1.71
	**Pestalotin**	1 (3)			12.4
	**Phenopyrrozin**	29 (97)	22.7 ± 18.1	16.8	2.43–89.3
	**Purpactin A**	5 (17)	5.03 ± 8.45	1.31	1.03–20.1
	**Questiomycin derivate**	13 (43)	18.3 ± 11.4	15.9	4.60–46.3
	**Quinolactacin A**	2 (7)	1.55 ± 0.66	1.55	1.08–2.01
	**Viridicatin**	1 (3)			1.17

^a^*n* = 30 cow’s total mixed ration (TMR) samples of dairy farms

^b^Samples with values > limit of detection (LOD), excluding data < LOD. In case of values > LOD and < limit of quantification (LOQ), LOQ/2 was used for calculation

Regarding *Penicillium*-derived metabolites, the most frequently detected were flavoglaucin (100%), phenopyrrozin (97%) and griseofulvin (73%). Flavoglaucin presented an average concentration of over 150 µg/kg and a maximum concentration > 1700 µg/kg. Other mycotoxins and metabolites derived from *Penicillium* spp. like OTA, OTB, mycophenolic acid and andrastin A occurred at low rates (< 30%) and low levels (70 µg/kg) (Table 2). Among the *Aspergillus*-produced metabolites, AFs’ precursors like averufin (70%), versicolorin C (63%), sterigmatocystin (STC) (37%) and seco-sterigmatocystin (27%) were found in levels ≤ 10 µg/kg in TMR samples. Kojic acid was detected in all samples and presented the highest levels (average: 134 µg/kg; max: 1060 µg/kg). Among *Alternaria* metabolites, tenuazonic acid and the mycoestrogens, alternariolmethylether and alternariol presented considerable occurrences of 97%, 90% and 40%, respectively. Tenuazonic acid was the *Alternaria* mycotoxin with the highest levels (average: 148 µg/kg; range: 71.8–492 µg/kg). Two ergot alkaloids were found: ergometrinine, which occurred in 73% of the samples, and ergosinine detected only in one sample. The levels of these toxic compounds were ≤ 10 µg/kg. Regarding metabolites derived from other fungal species, ascochlorin, cytochalasin D, ilicicolin B and neoechinulin A occurred at the rate of > 85%. Barceloneic acid was the fungal secondary metabolite with the highest concentration (average: 191 µg/kg; range: 52.8–588 µg/kg). Compounds like barceloneic acid, cercosporin, cytochalasin B, ilicicolin E and mollicellin D were detected in occurrences ranging from 20 to 50% (Table 2).

### Co-occurrence of mycotoxins and other secondary fungal metabolites

**Fig. 3:**
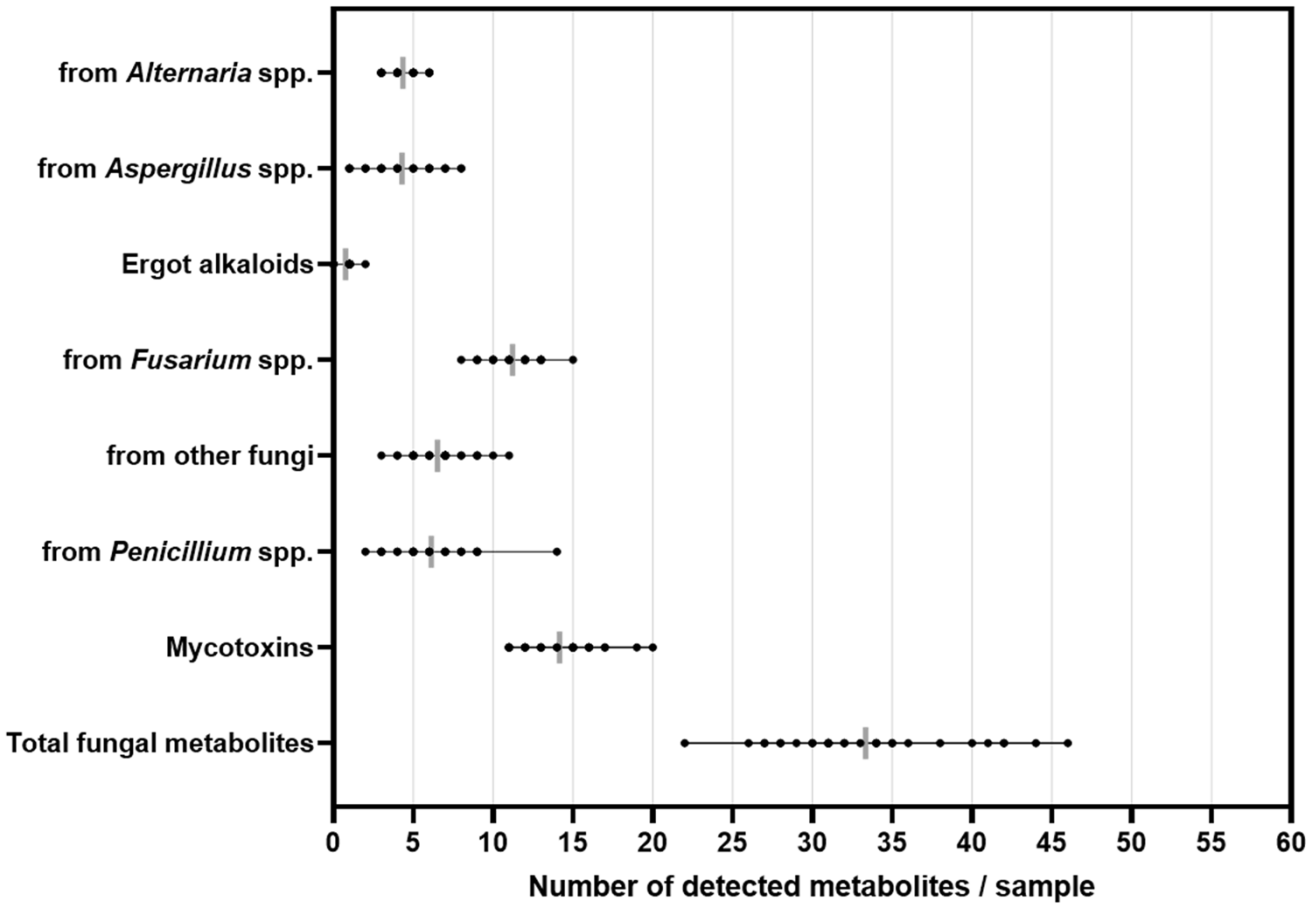
Scatter plots showing the co-contamination (number of metabolites/sample) in each metabolite group detected in the TMR samples from Punjab, Pakistan. The grey lines indicated the average numbers of detected metabolites per sample

**Fig. 4:**
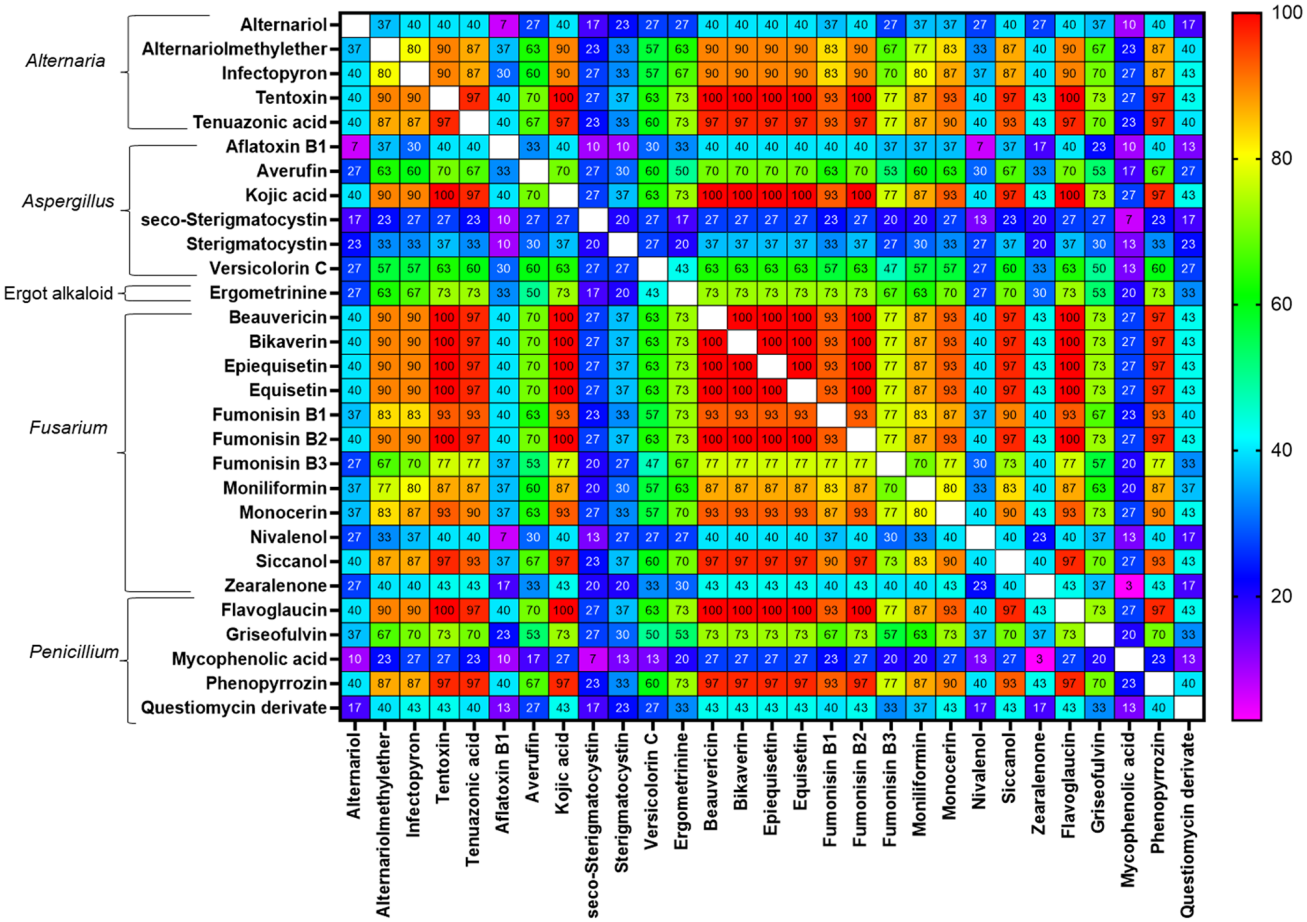
Heat map of the most frequent mycotoxin/metabolite combinations (in %) detected in the TMR samples (*n* = 30) from Punjab, Pakistan. Mycotoxins included in this analysis occurred in ≥ 30% of the samples

Figure 3 shows the average and distributions of co-contamination (i.e. the number of metabolites detected per sample) of different groups of metabolites. All TMRs were co-contaminated with several mycotoxins and other fungal metabolites. On average, 33 fungal metabolites per sample were detected, ranging from 22 to 46 fungal metabolites per sample. The mean number of mycotoxins per sample was 14, fluctuating from 11 to 20 mycotoxins per sample. On average, TMR contained 11 metabolites derived from *Fusarium* spp., fluctuating from eight to 15 metabolites per sample. Metabolites produced mainly by *Penicillium* spp. (mean: 6 metabolites per sample; range: 2 to 14 metabolites per sample) and from other fungi (7 metabolites per sample; range: 3 to 11 metabolites per sample), *Alternaria* spp. (4 metabolites per sample; range: 3 to 6 metabolites per sample) and *Aspergillus* spp. (4 metabolites per sample; range: 1 to 8 metabolites per sample) showed considerable levels of co-contamination (Fig. 3). The frequencies of co-occurrence analyses between mycotoxins and other fungal metabolites that occurred in > 30% of the samples are presented in Fig. 4. The most recurrent combinations (with co-occurrences over 90%) of detected metabolites in the TMR of dairy cows belonged to *Fusarium* spp. (like bikaverin, beauvericin, epiequisetin, equisetin, FB1 and FB2), *Alternaria* spp. (alternariolmethylether, infectopyrone, tentoxin and tenuazonic acid), *Aspergillus* spp. (kojic acid) and *Penicillium* spp. (phebopyrrozin) (Fig. 4).

### Relationship between concentrations and groups of mycotoxins and metabolites and the dietary ingredients

Positive moderate correlations (*ρ* > 0.5, *p* value < 0.001) were observed between corn grain, soybean meal and canola meal with FB1 and FB2 (Table 3). Also, the ergot alkaloid ergometrinine correlated positively with the content of molasses (*ρ* = 0.54, *p* value < 0.001). The proportion of commercial concentrate correlated negatively with the contamination levels of bikaverin (*ρ* = 0.54, *p* value < 0.001), FB1 (*ρ* =  − 0.56, *p* value < 0.001), FB3 (*ρ* =  − 0.50, *p* value < 0.001) and moniliformin (*ρ* =  − 0.56, *p* value < 0.001). All the values of the correlation analysis, i.e. *ρ* correlation coefficients and *p* values, are available in Supplementary Table S1. The moderate correlations between dietary ingredients and some of the toxins and metabolites (like FB1, FB2, bikaverin and ergometrinine) were confirmed by regression analyses (*p* value < 0.05) (Supplementary Fig. S1). Via regression analysis, a proportion of commercial concentrate showed no significant negative relationship with the levels of moniliformin (*p* value = 0.137).

**Table 3:** Spearman’s correlation among the proportions of dietary ingredients incorporated and levels of mycotoxins/fungal metabolites detected in total mixed rations of dairy farms in Punjab, Pakistan

**Mycotoxin/metabolite**	**Proportion of ingredient (% DM)**^a^						
	**Rhodes grass hay**	**Wheat straw**	**Corn grain**	**Soybean meal**	**Canola meal**	**Molasses**	**Commercial concentrate**
**Aflatoxin B1**	0.26	0.03	0.06	0.00	0.07	0.41*	− 0.26
**Kojic acid**	− 0.12	− 0.07	0.10	0.06	0.07	0.38*	− 0.23
**Ergometrinine**	− 0.23	0.26	0.39*	0.31	0.38*	0.54**	− 0.36
**Ergot alkaloids**	− 0.23	0.27	0.40*	0.31	0.38*	0.53**	− 0.35
**Bikaverin**	0.08	0.13	0.47*	0.47*	0.49**	0.31	− 0.58**
**Fumonisin B1**	− 0.16	0.22	0.54**	0.54**	0.52**	0.36	− 0.56**
**Fumonisin B2**	− 0.09	0.21	0.55**	0.56**	0.57**	0.32	− 0.47**
**Fumonisin B3**	0.02	0.18	0.47	0.42*	0.35	0.27	− 0.50**
**Moniliformin**	0.16	− 0.36	0.14	0.13	0.17	0.36	− 0.56**
**Neoechinulin A**	0.38*	− 0.26	− 0.12	− 0.03	− 0.06	0.03	− 0.40*
**Questiomycin derivate**	− 0.06	0.41*	− 0.29	− 0.23	− 0.19	− 0.12	− 0.12
**From *Penicillium***	0.23	0.10	− 0.19	− 0.18	− 0.11	0.32	− 0.05

^a^*n* = 29, one sampled farm declined to provide the information of the total mixed ration (TMR) composition

*p value < 0.05, significant; **p value < 0.01, highly significant

## Discussion

This study describes the mixtures of mycotoxins and other fungal metabolites in the complete diets of lactating cows at corporate dairy farms in Punjab, Pakistan. The region is considered a central crop-producing province and a crucial livestock-keeping area in the country (Younas and Yaqoob 2005; Akbar et al. 2019). The presented results again demonstrated the ubiquitous presence of mycotoxin mixtures in the complete diets of dairy cows. The mixtures fluctuated from 11 to 20 mycotoxins per ration. The cocktails of mycotoxins in commercial dairy cow farms have been previously revealed using multi-metabolite approaches (Awapak et al. 2021; Penagos-Tabares et al. 2022b). It is vital to note that this study is not representative for Pakistan, where most milk production and commercialization are informal. In the country, the formal sector (which sampled farms belong) has a small market share of merely 5%. Most dairy production and commercialization remain informal (Godfrey et al. 2018). Thus, public health’s additional investigation focused on Pakistan’s informal dairy chain is highly advised.

This research confirmed (as expected) the presence of AFB1. This toxin, produced mainly by *Aspergillus* spp., is the most toxic and recurrent among the AFs and is the most potent natural hepatocarcinogenic agent in mammals. It is classified as a group 1 human carcinogen by the International Agency for Research on Cancer (IARC 2012; Marchese et al. 2018). AFB1 was detected in around 40% of the rations in the current study. This situation implicates the AFM1 contamination of milk in some of these farms, which is a global issue, particularly in developing countries (Groopman et al. 2008). AFB1 concentrations exceeding the maximum level of the EU were detected in 7% of the TMR. Additionally, precursors of AFs, such as averufin, STC and versicolorin C (Cary et al. 2006; Hsieh et al. 1973), were detected in frequencies < 35%. Regarding AFs and STC, it has been suggested that these mycotoxins can be produced pre- and post-harvest (Mo et al. 2015). Like AFs, STC is known to be carcinogenic with immunotoxin and immunomodulatory activity. The information available on exposure data of dairy cows and other animals to STC is limited (EFSA 2013; Gruber-Dorninger et al. 2017; Chuang et al. 2020). One sample was contaminated with AFM1 (1.18 µg/kg), and AFM1 is not found in plants, a fact that indicates the contamination with animal products, particularly milk or dairy products mixed in the TMR (Min et al. 2021).

Aflatoxicosis in cattle includes clinical signs such as poor weight gains, decreased feed conversion and milk production, lethargy, inappetence, ataxia and increment of hepatic enzymes and bilirubin, in addition to prolonged clotting times (Diekman and Green 1992). Cows fed with diets containing AFB1 at concentrations of 20 µg/kg presented a depletion in the feed intake and milk yield. Three days after the source of AFB1 was removed, the clinical signs began to improve (Jones and Ewart 1979). Similarly, another field study, which assessed the effect of aflatoxin-contaminated corn on lactating dairy cattle, observed a decline in reproductive efficiency. After the inclusion of an aflatoxin-free diet, an increment of 25% of the milk yield was evidenced (Guthrie and Bedell 1979) as cited by Jouany and Diaz (2005). Several case reports of acute aflatoxicosis in cattle have been described. For example, a group of crossbred feeder steers fed with corn contaminated with aflatoxin at a concentration of 1.5 µg/kg generated typical hepatic lesions. Mycotoxin residues were detected in the kidney tissue (Colvin et al. 1984). In the same way, a small herd of cattle having access to mouldy and unharvested sweet corn revealed via postmortem examinations oedema of all soft tissues and liver lesions consistent with aflatoxicosis. Weather conditions were favourable for the proliferation of *Aspergillus flavus* and *Aspergillus parasiticus*, and the contamination levels of the corn samples taken from the field contained 2365 ng of aflatoxin/g (Hall et al. 1989). Minimizing AFB1 contamination in dairy feeds needs good agricultural and management practices at pre-harvest stages, such as appropriate harvest time, maintenance of crop health by avoiding pest infestations and use of fungal-resistant varieties of crops. At post-harvest, the reduction of moisture for conserved feedstuffs, proper storage at low temperature and humidity and protection against pest infestation (by insecticides and fungicides) are advocated. Also, routine monitoring for aflatoxins in feeds using aflatoxin binders/inactivators in feed and creating awareness among farmers on the health impacts of aflatoxins have been proposed to reduce risks (Patyal et al. 2021).

Mycotoxins from *Fusarium* (e.g. FB1, FB2, beauvericin and bikaverin), *Alternaria* (e.g. alternariolmethylether and tenuazonic acid) and *Aspergillus* (e.g. kojic acid, averufin and STC) along with *Penicillium* toxins (like mycophenolic acid) and other metabolites were recently reported in diets of dairy cattle in Thailand and Austria (Awapak et al. 2021; Penagos-Tabares et al. 2022, survey). Like in European dairy cattle diets (Penagos-Tabares et al. 2022b), the current study found *Fusarium* mycotoxins/metabolites as a dominant group of fungal metabolites. The occurrences and levels of ZEN, enniatins and ergot alkaloids were lower than those of diets of Austrian dairy cattle (Penagos-Tabares et al. 2022b), and fumonisin contamination was higher in the dairy diets of Pakistan (100% of the samples contaminated with at least one fumonisin). Our results also highlight the role of tenuazonic acid as the most abundant mycotoxin produced by *Alternaria* spp.; however, the information regarding the occurrence and toxic effects of these toxins in animals is still scarce and, therefore, health risks associated with *Alternaria* toxins in feeds have not yet been clarified (EFSA 2011). Regarding the occurrence of trichothecenes, with an occurrence of 40%, NIV was the only mycotoxin of this group detected. A study reported NIV as the most occurrent trichothecene (12.3%), which was usually detected co-occurring with other trichothecenes like DON, T-2, HT-2 and 3-acetyl-deoxynivalenol in maize grain in Punjab, Pakistan. However, the study also reports samples contaminated only with NIV, which suggest the presence of NIV-dominant *Fusarium* chemotypes (Khatoon et al. 2012).

Various *Penicillium*-derived compounds have been previously detected in silages, such as mycophenolic acid, mycophenolic acid IV and andrastin A (Gallo et al. 2015; Penagos-Tabares et al. 2022a, b; Storm et al. 2014). OTA, contemplated in the European regulation, is produced by species of *Penicillium* and *Aspergillus* and presented a low occurrence and contamination levels, which suggest that this mycotoxin presents a negligible risk for dairy herds, in line with previous studies (Driehuis et al. 2008; Awapak et al. 2021; Penagos-Tabares et al. 2022b). Additionally, produced primarily by *Aspergillus* spp., but by *Penicillium* and *Acetobacter* fungi (Parrish et al. 1966), kojic acid has shown low toxicity for human macrophages and antibacterial and immunomodulatory properties (Morton et al. 1945; Kotani et al. 1976; Bashir et al. 2021). Additionally, further less-known metabolites are produced by other fungi detected in the dairy cows’ diets. Some of them have antibacterial activity, for example the illicicolins (Hayakawa et al. 1971), cytochalasins (Aldridge et al. 1967; Jouda et al. 2016) and ascochlorin also known as antibiotic LL-Z1272γ and ilicicolin D (Molnár et al. 2010). The current results showed tenuazonic acid as the most abundant mycotoxin produced by *Alternaria* spp.; however, the information regarding the occurrence and toxic effects of these toxins in animals is still scarce and, therefore, health risks associated with *Alternaria* toxins in feeds have not been elucidated (EFSA 2011).

The critical factors facilitating the growth of aflatoxin-producing moulds in corn grains and silage include, among others, lack of good agricultural storage practices and unfavourable climatic conditions (Kebede et al. 2012; Frazzoli et al. 2016). The risk of aflatoxin contamination is generally higher in geographical regions with a tropical climate or a sub-tropical climate, but an extremely hot and droughty season may promote the growth of *Aspergillus* spp. in crops (Kebede et al. 2012). AFB1 has been reported in dairy feeds in Thailand, with an occurrence of 39% in concentrate (Awapak et al. 2021). European reports are rare; however, 61% of the TMR from Lithuanian dairy farms tested positive for AFB1 (mean: 2.42 µg/kg, range: 1.03–5.00 µg/kg) (Vaičiulienė et al. 2021). The incidence of TMRs reported was 90% in Spain (Hernandez-Martinez and Navarro-Blasco 2015) and 8.1% in Italy (Decastelli et al. 2007). A moderate positive correlation of molasses with ergot alkaloids (specifically with ergometrinine) can be explained because ergot can grow on sugarcane (*Saccharum* spp.) (Singh 1976). Molasses is dehydrated sugarcane juice; some ergot alkaloids could be found in high concentration in molasses due to its concentration during fabrication and the thermostability of the ergot alkaloids.

Different metabolite profiles could result from the same genus and species depending on the high variability of strains, substrate and growing conditions (Daou et al. 2021). The diversity of mycotoxins and fungal secondary metabolites is due to the multi-commodity composition of the diets. Despite the risk associated with toxicological interactions of mycotoxins, there is hardly any regulation on their combined occurrence globally (Battilani et al. 2020; Singh 2022). This study evidenced the high occurrence of a broad number of mycotoxins (most of them not contemplated in the legislation) and other fungal secondary metabolites occurring in dairy TMR in Pakistan. Around 7% of the samples exceeded the GVs of the EU commission for AFB1. Moreover, a vast majority of mycotoxins and metabolites are emerging, as well as less-known and less-studied fungal metabolites. After the compounds derived from other fungal species were analysed, it was observed that *Fusarium*-produced metabolites and mycotoxins were the dominant fungal contaminants. Additionally, the data derived from Spearman’s correlation test (Table 3 and Table S1) and lineal regressions (Fig. S1) show consistently that moderate positive relationships among the dietary contents of ingredients like corn grain, soybean meal and canola meal were related to increased contamination of some *Fusarium* mycotoxins (like FB1, FB2 and FB3) in the TMR from the province of Punjab, Pakistan. Considering the low sample size of this exploratory study, both statistical methods (correlations and linear regression) were used to explore the relationships of ration formulation (ingredients) with mycotoxin/metabolite concentrations. In contrast with studies in other regions like South America and Europe (Driehuis et al. 2008; Signorini et al. 2012; Reisinger et al. 2019; Penagos-Tabares et al. 2022a), our results do not reveal maize silage as one of the most influential feedstuffs to the mycotoxin/metabolite contamination. Among the typical forage sources, the content of maize silage was ubiquitous in the analysed rations. However, a previous study suggested the role of cottonseed cake as the contributor to around 80% of the AFB1 in diets of dairy cattle in periurban farms in Punjab (Yunus et al. 2020). It is also crucial to consider that more consistent association and relationship assessments would require a higher sample size.

Except for AFB1, which represents a risk for animals and human consumers due to AFM1 content in milk (Min et al. 2021), other detected mycotoxins correspond to a relatively low level of risk. However, the realistic scenario, the long-term exposure to multiple mycotoxins and other fungal secondary metabolites could have unpredictable effects on animal health, reproduction and productivity. However, the high co-occurrence of various mycotoxins/metabolites should be investigated because of their potential toxicological interactions (additive or synergistic effects) and long-term effects at low chronic exposure (Smith et al. 2016; Battilani et al. 2020). At the detected levels, no other mycotoxin than AFB1 was reported to have a considerable transfer of metabolites into milk and other animal products. The findings suggest that it is necessary to design effective strategies to verify the safety of feedstuffs utilized in ration formulation. More surveillance and further research based on multi-metabolite methodologies in the dairy industry in other geographic regions of Pakistan and the world, considering seasonal variation, are still strongly encouraged. More governmental interest and research are essential for this concern to ensure the offer of safe dairy products to the consumer and support animal health and the productive potential of dairy herds.

This exploratory study evidenced that the most relevant mycotoxin for public health, the carcinogenic AFB1, is occurring in diets of big commercial dairy farms (> 200 lactating dairy cows) in the province of Punjab, Pakistan. AFB1 was detected in concentrations seven times higher than the EU maximum limit, representing a severe risk to animal health and human milk consumers. No other mycotoxin than AFB1 exceeded the EU guidance values. Except for ergot alkaloids, all the groups of metabolites (i.e. derived from *Alternaria* spp., *Aspergillus* spp., *Fusarium* spp., *Penicillium* spp. and other fungi) occurred in 100% of the TMR samples. Although the detected contamination levels of single compounds (mycotoxins/metabolites) are moderately low, the effects on animal health, reproduction and productivity under the detected realistic scenario (“cocktails effect”) are still unpredictable. Similar studies with higher sample size and approaching other regions are extremely advocated. Thus, future toxicological studies should address such interactions (additivity, potentiation, synergism and antagonism), as well as the long-term exposure effects of “mycotoxin mixtures”. The presented results reconfirm that the monitoring and surveillance of aflatoxin M1 in dairy products in the South Asian region are essential and highly required.

### Supplementary Information

Below is the link to the electronic supplementary material.

Supplementary file1 (PDF 683 KB)

## Data Availability

Software application or custom code.

## References

[CR1] Akbar N, Nasir M, Naeem N, Ahmad MD, Iqbal S, Rashid A, Imran M, Gondal TA, Atif M, Salehi B, Sharifi-Rad J, Martorell M, Cho WC (2019). Occurrence and seasonal variations of aflatoxin M1 in milk from Punjab. Pakistan Toxins.

[CR2] Akbar N, Nasir M, Naeem N, Ahmad MD, Saeed F, Anjum FM, Iqbal S, Imran M, Tufail T, Shah FH, Atif M (2020). Assessment of aflatoxin in milk and feed samples and impact of seasonal variations in the Punjab, Pakistan. Food Sci Nutr.

[CR3] Aldridge D, Armstrong J, Speake R, Turner W (1967). The cytochalasins, a new class of biologically active mould metabolites. Chem Commun.

[CR4] Ashiq S (2015). Natural occurrence of mycotoxins in food and feed: Pakistan perspective. Compr Rev Food Sci Food Saf.

[CR5] Aslam N, Wynn PC (2015). Aflatoxin contamination of the milk supply: a Pakistan perspective. Agriculture.

[CR6] Awapak D, Petchkongkaew A, Sulyok M, Krska R (2021). Co-occurrence and toxicological relevance of secondary metabolites in dairy cow feed from Thailand. Food Addit Contam Part A Chem Anal Control Expo Risk Assess.

[CR7] Bashir F, Sultana K, Khalid M, Rabia H (2021). Kojic Acid: a Comprehensive Review AJAHS.

[CR8] Battilani P, Palumbo R, Giorni P, Dall’Asta C, Dellafiora L, Gkrillas A, Toscano P, Crisci A, Brera C, De Santis B, Cammarano RR, Della Seta M, Campbell K, Elliot C, Venancio A, Lima N, Gonçalves A, Terciolo C, Oswald IP (2020) Mycotoxin mixtures in food and feed: holistic, innovative, flexible risk assessment modelling approach: MYCHIF. EFSA Support Publ 17:1757E. Available from: 10.2903/sp.efsa.2020.EN-1757. Accessed 5 Aug 2022

[CR9] Bryden WL (2012). Mycotoxin contamination of the feed supply chain: implications for animal productivity and feed security. Anim Feed Sci Technol.

[CR10] Bueno AVI, Lazzari G, Jobim CC, Daniel JLP (2020). Ensiling total mixed ration for ruminants: a review. Agronomy.

[CR11] Cary JW, Ehrlich KC, Bland JM, Montalbano BG (2006) The aflatoxin biosynthesis cluster gene, aflX, encodes an oxidoreductase involved in conversion of versicolorin A to demethylsterigmatocystin. Appl Environ Microbiol 72:1096–1101. 10.2903/sp.efsa.2020.EN-1757. Accessed 5 Aug 2022 10.1128/AEM.72.2.1096-1101.2006 PMC1392920 16461654

[CR12] Chuang WY, Hsieh YC, Lee T-T (2020) The effects of fungal feed additives in animals: a review. Animals 10(5):805. 10.3390/ani10050805 10.3390/ani10050805 PMC7278461 32384791

[CR13] Cinar A, Onbaşı E (2019) Mycotoxins: the hidden danger in foods. In: Mycotoxins and food safety: IntechOpen, London

[CR14] Codex Alimentarius Commission (2001) Comments submitted on the draft maximum level for aflatoxin M1 in milk. Codex Committee on Food Additives and Contaminants 33rd Session, Hague, The Netherlands

[CR15] Colvin B, Harrison L, Gosser H, Hall R (1984). Aflatoxicosis in feeder cattle. J Am Vet Med Assoc.

[CR16] Daou R, Joubrane K, Maroun RG, Khabbaz LR, Ismail A, El Khoury A (2021). Mycotoxins: factors influencing production and control strategies. AIMS Agric Food.

[CR17] Decastelli L, Lai J, Gramaglia M, Monaco A, Nachtmann C, Oldano F, Ruffier M, Sezian A, Bandirola C (2007). Aflatoxins occurrence in milk and feed in Northern Italy during 2004–2005. Food Control.

[CR18] Diekman MA, Green ML (1992). Mycotoxins and reproduction in domestic livestock. J Anim Sci.

[CR19] Driehuis F, Spanjer MC, Scholten JM, Giffel MCT (2008). Occurrence of mycotoxins in feedstuffs of dairy cows and estimation of total dietary intakes. J Dairy Sci.

[CR20] EC – European Commission (2002) Directive 2002/32/EC of the European Parliament and of the Council of 7 May 2002 on undesirable substances in animal feed. Luxemb Off J Eur Union 140:10–22. Last consolidated version available from: https://eur-lex.europa.eu/legal-content/EN/TXT/?uri=CELEX%3A02002L0032-20191128. Accessed 5 Aug 2022

[CR21] EC – European Commission (2006) Commission Recommendation (EU) 2006/576/EC of 17 August 2006 on the presence of deoxynivalenol, zearalenone, ochratoxin A, T-2 and HT-2 and fumonisins in products intended for animal feeding. Off J Eur Union, 229, 7–9. Last consolidated version available from: https://eur-lex.europa.eu/legal-content/EN/TXT/HTML/?uri=CELEX:02006H0576-20160802&from=EN. Accessed 5 Aug 2022

[CR22] EFSA – European Food Safety Authority, Panel on Contaminants in the Food Chain (2011) Scientific Opinion on the risks for animal and public health related to the presence of *Alternaria* toxins in feed and food. EFSA J 9:2407. Available from: 10.2903/j.efsa.2011.2407. Accessed 5 Aug 2022

[CR23] EFSA – European Food Safety Authority, Panel on Contaminants in the Food Chain (2013) Scientific Opinion on the risk for public and animal health related to the presence of sterigmatocystin in food and feed. EFSA J 11:3254. Available from: 10.2903/j.efsa.2013.3254. Accessed 5 Aug 2022

[CR24] FAO – Food and Agriculture Organization of the United Nations (2021) Overview of global dairy market developments in 2020, Rome. Available from: https://www.fao.org/publications/card/en/c/CB4230EN/

[CR25] FAO, IDF, IFCN – Food and Agriculture Organization of the United Nations, International Dairy Federation, Dairy Research Network (2014) World mapping of animal feeding systems in the dairy sector. FAO, Rome. Available from: https://www.fao.org/3/i3913e/i3913e.pdf. Accessed 5 Aug 2022

[CR26] Frazzoli C, Gherardi P, Saxena N, Belluzzi G, Mantovani A (2016). The hotspot for (global) one health in primary food production: aflatoxin M1 in dairy products. Front Public Health.

[CR27] Gallo A, Giuberti G, Frisvad JC, Bertuzzi T, Nielsen KF (2015). Review on mycotoxin issues in ruminants: occurrence in forages, effects of mycotoxin ingestion on health status and animal performance and practical strategies to counteract their negative effects. Toxins.

[CR28] Godfrey SS, Ramsay GC, Behrendt K, Wynn PC, Nordblom TL, Aslam N (2018) Analysis of agribusiness value chains servicing small-holder dairy farming communities in Punjab, Pakistan: three case studies. Int Food Agribus Manag Rev 22:119–136. 10.22434/IFAMR2017.0122

[CR29] Groopman JD, Kensler TW, Wild CP (2008). Protective interventions to prevent aflatoxin-induced carcinogenesis in developing countries. Annu Rev Public Health.

[CR30] Gruber-Dorninger C, Jenkins T, Schatzmayr G (2019). Global mycotoxin occurrence in feed: a ten-year survey. Toxins.

[CR31] Gruber-Dorninger C, Novak B, Nagl V, Berthiller F (2017). Emerging mycotoxins: beyond traditionally determined food contaminants. J Agric Food Chem.

[CR32] Guthrie L, Bedell D (1979). Effects of aflatoxin in corn on production and reproduction in dairy cattle. Proc Annu Meet US Anim Health Assoc.

[CR33] Hajnal EJ, Kos J, Malachová A, Steiner D, Stranska M, Krska R, Sulyok M (2020) Mycotoxins in maize harvested in Serbia in the period 2012–2015. Part 2: non-regulated mycotoxins and other fungal metabolites. Food Chem 317:126409. 10.1016/j.foodchem.2020.126409 10.1016/j.foodchem.2020.126409 32087516

[CR34] Hall R, Harrison L, Colvin B (1989). Aflatoxicosis in cattle pastured in a field of sweet corn. J Am Vet Med Assoc.

[CR35] Hayakawa S, Minato H, Katagiri K (1971). The ilicicolins, antibiotics from *Cylindrocladium ilicicola*. J Antibiot.

[CR36] Hernandez-Martinez R, Navarro-Blasco I (2015). Surveillance of aflatoxin content in dairy cow feedstuff from Navarra (Spain). Anim Feed Sci Technol.

[CR37] Hinkle DE, Wiersma W, Jurs SG (2003) Applied statistics for the behavioral sciences (Vol. 663): Houghton Mifflin College Division, Boston

[CR38] Hsieh D, Lin M, Yao R (1973). Conversion of sterigmatocystin to aflatoxin B1 by *Aspergillus parasiticus*. Biochem Biophys Res Commun.

[CR39] Hussain I, Anwar J (2008). A study on contamination of aflatoxin M1 in raw milk in the Punjab Province of Pakistan. Food Control.

[CR40] IARC – International Agency for Research on Cancer (2012) Agents classified by the IARC Monographs, volumes 1–106. http://monographs.iarc.fr/ENG/Classification/index.php.

[CR41] Iqbal SZ, Asi MR (2013). Assessment of aflatoxin M1 in milk and milk products from Punjab, Pakistan. Food Control.

[CR42] Jones M, Ewart J (1979). Effects on milk production associated with consumption of decorticated extracted groundnut meal contaminated with aflatoxin. Vet Rec.

[CR43] Jouany JP, Diaz DE, Diaz DE (2005). Effects of mycotoxins in ruminants. The mycotoxin blue book.

[CR44] Jouda JB, Tamokou JdD, Mbazoa CD, Douala-Meli C, Sarkar P, Bag PK, Wandji J (2016). Antibacterial and cytotoxic cytochalasins from the endophytic fungus *Phomopsis* sp. harbored in *Garcinia kola* (Heckel) nut. BMC Complement Altern Med.

[CR45] Kebede H, Abbas HK, Fisher DK, Bellaloui N (2012). Relationship between aflatoxin contamination and physiological responses of corn plants under drought and heat stress. Toxins.

[CR46] Khatoon S, Hanif NQ, Tahira I, Sultana N, Sultana K, Ayub N (2012). Natural occurrence of aflatoxins, zearalenone and trichothecenes in maize grown in Pakistan. Pak J Bot.

[CR47] Kotani T, Ichimoto I, Tatsumi C, Fujita T (1976). Bacteriostatic activities and metal chelation of kojic acid analogs. Agric Biol Chem.

[CR48] Marchese S, Polo A, Ariano A, Velotto S, Costantini S, Severino L (2018). Aflatoxin B1 and M1: biological properties and their involvement in cancer development. Toxins.

[CR49] Massey TE, Stewart RK, Daniels JM, Liu L (1995). Biochemical and molecular aspects of mammalian susceptibility to aflatoxin B1 carcinogenicity. Proc Soc Exp Biol Med.

[CR50] Min L, Fink-Gremmels J, Li D, Tong X, Tang J, Nan X, Yu Z, Chen W, Wang G (2021). An overview of aflatoxin B1 biotransformation and aflatoxin M1 secretion in lactating dairy cows. Anim Nutr.

[CR51] Mo HG, Pietri A, MacDonald SJ, Anagnostopoulos C, Spanjere M (2015) Survey on sterigmatocystin in food. EFSA Support Publ 12:774E. Available from: 10.2903/sp.efsa.2015.EN-774

[CR52] Molnár I, Gibson DM, Krasnoff SB (2010). Secondary metabolites from entomopathogenic Hypocrealean fungi. Nat Prod Rep.

[CR53] Morton HE, Kocholaty W, Junowicz-Kocholaty R, Kelner A (1945). Toxicity and antibiotic activity of kojic acid produced by *Aspergillus luteo-virescens*. J Bacteriol.

[CR54] Nichea MJ, Cendoya E, Zachetti VGL, Chiacchiera SM, Sulyok M, Krska R, Torres AM, Chulze SN, Ramirez ML (2015). Mycotoxin profile of *Fusarium armeniacum* isolated from natural grasses intended for cattle feed. World Mycotoxin J.

[CR55] Nichea MJ, Palacios SA, Chiacchiera SM, Sulyok M, Krska R, Chulze SN, Torres AM, Ramirez ML (2015). Presence of multiple mycotoxins and other fungal metabolites in native grasses from a wetland ecosystem in Argentina intended for grazing cattle. Toxins.

[CR56] Panasiuk L, Jedziniak P, Pietruszka K, Piatkowska M, Bocian L (2019). Frequency and levels of regulated and emerging mycotoxins in silage in Poland. Mycotoxin Res.

[CR57] Parrish F, Wiley B, Simmons E, JrL L (1966). Production of aflatoxins and kojic acid by species of Aspergillus and Penicillium. Appl Microbiol.

[CR58] Patyal A, Gill JPS, Bedi JS, Aulakh RS (2021). Assessment of aflatoxin contamination in dairy animal concentrate feed from Punjab, India. Environ Sci Pollut Res.

[CR59] Penagos-Tabares F, Khiaosa-ard R, Nagl V, Faas J, Jenkins T, Sulyok M, Zebeli Q (2021). Mycotoxins, phytoestrogens and other secondary metabolites in Austrian pastures: occurrences, contamination levels and implications of geo-climatic factors. Toxins.

[CR60] Penagos-Tabares F, Khiaosa-ard R, Schmidt M, Bartl EM, Kehrer J, Nagl V, Faas J, Sulyok M, Krska R, Zebeli Q (2022). Cocktails of mycotoxins, phytoestrogens, and other secondary metabolites in diets of dairy cows in Austria: inferences from diet composition and geo-climatic factors. Toxins.

[CR61] Penagos-Tabares F, Khiaosa-Ard R, Schmidt M, Pacífico C, Faas J, Jenkins T, Nagl V, Sulyok M, Labuda R, Zebeli Q (2022). Fungal species and mycotoxins in mouldy spots of grass and maize silages in Austria. Mycotoxin Res.

[CR62] Rasmussen RR, Storm I, Rasmussen PH, Smedsgaard J, Nielsen KF (2010). Multi-mycotoxin analysis of maize silage by LC-MS/MS. Anal Bioanal Chem.

[CR63] Reisinger N, Schurer-Waldheim S, Mayer E, Debevere S, Antonissen G, Sulyok M, Nagl, V. (2019) Mycotoxin occurrence in Maize Silage—A neglected risk for bovine gut health? Toxins 11(10):577. 10.3390/toxins11100577 10.3390/toxins11100577 PMC6832361 31590302

[CR64] Sadia A, Jabbar MA, Deng Y, Hussain EA, Riffat S, Naveed S, Arif M (2012). A survey of aflatoxin M1 in milk and sweets of Punjab, Pakistan. Food Control.

[CR65] Santos Pereira C, Cunha SC, Fernandes JO (2019). Prevalent mycotoxins in animal feed: occurrence and analytical methods. Toxins.

[CR66] Santos RR, Fink-Gremmels J (2014). Mycotoxin syndrome in dairy cattle: characterisation and intervention results. World Mycotoxin J.

[CR67] Schingoethe DJ (2017). A 100-year review: total mixed ration feeding of dairy cows. J Dairy Sci.

[CR68] Shimshoni JA, Cuneah O, Sulyok M, Krska R, Galon N, Sharir B, Shlosberg A (2013). Mycotoxins in corn and wheat silage in Israel. Food Addit Contam Part A Chem Anal Control Expo Risk Assess.

[CR69] Signorini ML, Gaggiotti M, Molineri A, Chiericatti CA, de Basilico MLZ, Basilico JC, Pisani M (2012). Exposure assessment of mycotoxins in cow’s milk in Argentina. Food Chem Toxicol.

[CR70] Singh K, Kumari A (2022) Mycotoxins co-occurrence poisoning. In: Mycotoxins and mycotoxicoses. Springer, Singapore. 10.1007/978-981-19-2370-8_6

[CR71] Singh S (1976) Occurrence of ergot and false floral smut on *Saccharum spontaneum* in India. J Plant Dis Prot 83(7/8):442–447. http://www.jstor.org/stable/43214102

[CR72] Smith MC, Madec S, Coton E, Hymery N (2016). Natural co-occurrence of mycotoxins in foods and feeds and their in vitro combined toxicological effects. Toxins.

[CR73] Steiner D, Sulyok M, Malachová A, Mueller A, Krska R (2020) Realizing the simultaneous liquid chromatography-tandem mass spectrometry based quantification of > 1200 biotoxins, pesticides and veterinary drugs in complex feed. J Chromatogr A 1629:461502. 10.1016/j.chroma.2020.461502 10.1016/j.chroma.2020.461502 32841773

[CR74] Storm I, Rasmussen RR, Rasmussen PH (2014). Occurrence of pre- and post-harvest mycotoxins and other secondary metabolites in Danish maize silage. Toxins.

[CR75] Sulyok M, Stadler D, Steiner D, Krska R (2020). Validation of an LC-MS/MS-based dilute-and-shoot approach for the quantification of > 500 mycotoxins and other secondary metabolites in food crops: challenges and solutions. Anal Bioanal Chem.

[CR76] Szulc J, Okrasa M, Dybka-Stępień K, Sulyok M, Nowak A, Otlewska A, Szponar B, Majchrzycka K (2019). Assessment of microbiological indoor air quality in cattle breeding farms. AAQR.

[CR77] Vaičiulienė G, Bakutis B, Jovaišienė J, Falkauskas R, Gerulis G, Kerzienė S, Baliukonienė V (2021). Prevalence of mycotoxins and endotoxins in total mixed rations and different types of ensiled forages for dairy cows in Lithuania. Toxins.

[CR78] Younas M, Yaqoob M (2005). Feed resources of livestock in the Punjab. Pakistan Livest Res Rural Dev.

[CR79] Yunus AW, Ullah A, Lindahl JF, Anwar Z, Ullah A, Saif S, Ali M, Zahur AB, Irshad H, Javaid S, Imtiaz N, Farooq U, Ahsan A, Zahida F, Hashmi AA, Abbasi BHA, Bari Z, Khan IU, Ibrahim MNM (2020). Aflatoxin contamination of milk produced in peri-urban farms of Pakistan: prevalence and contributory factors. Front Microbiol.

