# Supplementary material for: Co-occurrence of mycotoxins and other fungal metabolites in total mixed rations of cows from dairy farms in Punjab, Pakistan
Source: Mycotoxin Res. 2023 Sep 4;39(4):421–36. doi: 10.1007/s12550-023-00502-5 (PMC10635927; doi:10.1007/s12550-023-00502-5)
Supplement: Supplementary file 1 — Supplementary file1 (PDF 683 KB) [file 12550_2023_502_MOESM1_ESM.pdf]

Supplementary Fig. S1

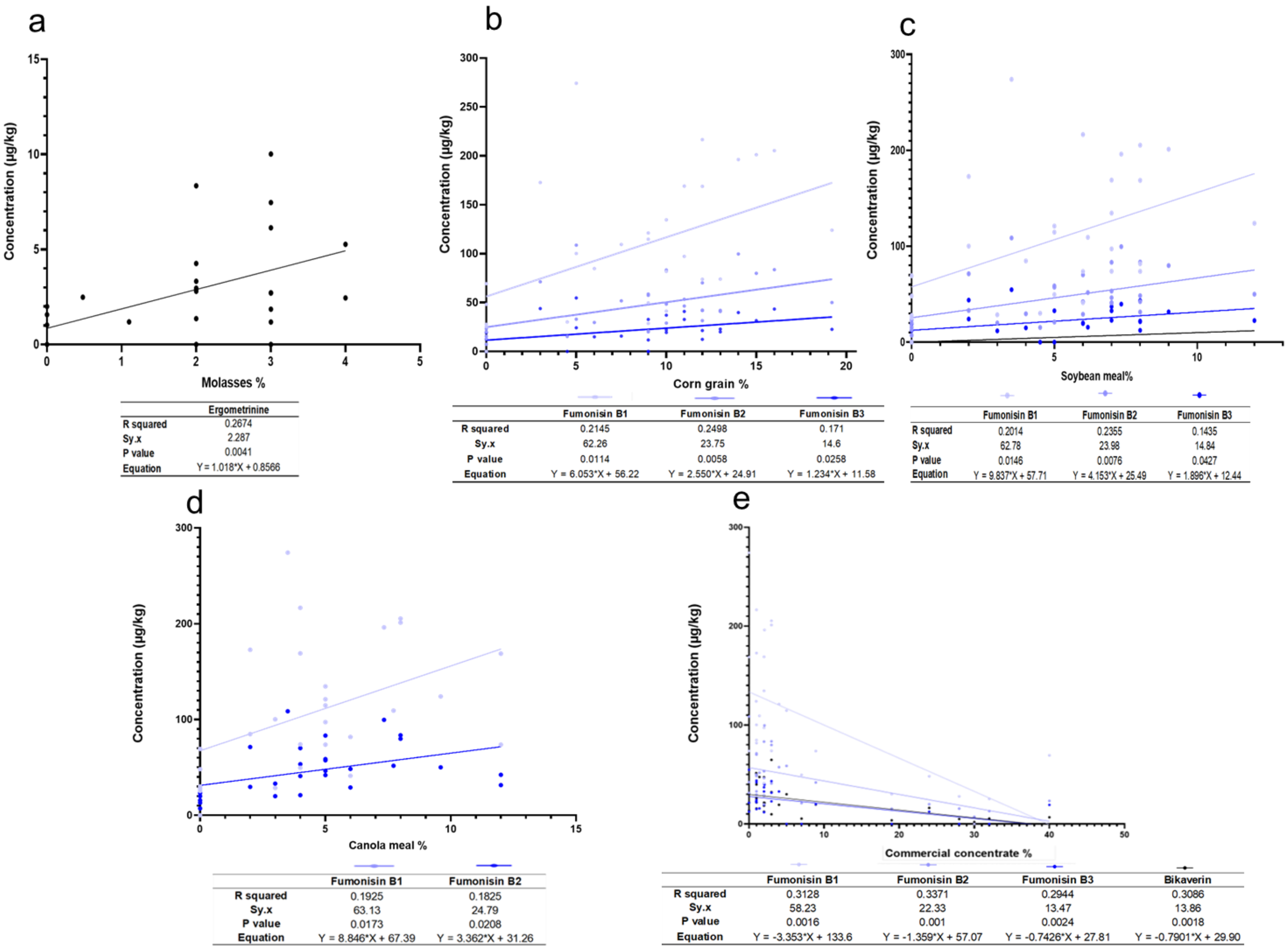

Table S1. Spearman correlation among the proportion of dietary ingredients incorporated and levels of mycotoxins/fungal metabolites detected in total mixed rations of dairy farms from Punjab, Pakistan. ρ (Rho): Correlation coefficient.

	ρ	P value	ρ	P value	P	P value	ρ	P value	ρ	P value	ρ	P value	ρ	P value	ρ	P value	ρ	P value	ρ	P value
	Corn silage %	Corn silage %	Rhodes grass hay%	Rhodes grass hay%	Wheat straw%	Wheat straw%	Corn grain %	Corn grain %	Soybean meal%	Soybean meal%	Canola meal %	Canola meal %	Molasses %	Molasses %	Commercial concentrate %	Commercial concentrate %	Concentrate %	Concentrate %	Forage %	Forage %
Alternariolmethylether	-0.13	0.5016	-0.24	0.2116	0.15	0.4289	-0.08	0.6837	-0.05	0.8063	-0.18	0.3509	-0.11	0.5865	0.04	0.8262	0.13	0.5020	-0.13	0.5020
Infectopyron	-0.11	0.5702	-0.19	0.3222	0.33	0.0758	-0.17	0.3677	-0.15	0.4260	-0.09	0.6287	-0.26	0.1775	0.31	0.1054	0.17	0.3825	-0.17	0.3825
Tentoxin	-0.07	0.7287	0.18	0.3638	-0.27	0.1560	-0.47	0.0101	-0.41	0.0278	-0.25	0.1955	-0.02	0.9062	-0.14	0.4849	-0.12	0.5445	0.12	0.5445
Tenuazonic acid	0.07	0.7025	-0.55	0.0019	0.28	0.1433	-0.24	0.2169	-0.19	0.3331	-0.15	0.4235	-0.08	0.6645	-0.01	0.9512	0.03	0.8676	-0.03	0.8676
from *Alternaria*	-0.08	0.6799	-0.51	0.0046	0.36	0.0584	-0.23	0.2347	-0.20	0.2946	-0.16	0.4063	-0.20	0.3005	0.21	0.2833	0.17	0.3771	-0.17	0.3771
Aflatoxin B1	-0.06	0.7585	0.26	0.1802	0.03	0.8882	0.06	0.7449	0.00	0.9886	0.07	0.7106	0.41	0.0259	-0.26	0.1760	-0.32	0.0958	0.32	0.0958
Averufin	0.21	0.2837	0.07	0.7298	0.01	0.9524	-0.24	0.2005	-0.11	0.5840	-0.20	0.2925	-0.30	0.1093	0.32	0.0879	-0.02	0.9284	0.02	0.9284
Kojic acid	-0.16	0.3925	-0.12	0.5490	-0.07	0.7110	0.10	0.5906	0.06	0.7447	0.07	0.7373	0.38	0.0437	-0.23	0.2354	-0.02	0.9189	0.02	0.9189
Sterigmatocystin	-0.07	0.7290	0.07	0.7209	-0.06	0.7667	0.02	0.9000	0.18	0.3484	0.12	0.5216	-0.16	0.4187	0.12	0.5377	0.21	0.2662	-0.21	0.2662
Versicolorin C	0.21	0.2829	-0.14	0.4778	-0.07	0.7208	-0.27	0.1522	-0.07	0.7071	-0.21	0.2739	-0.39	0.0389	0.18	0.3463	0.01	0.9473	-0.01	0.9473
from *Aspergillus*	-0.14	0.4752	-0.10	0.6184	-0.10	0.6003	0.06	0.7401	0.06	0.7739	0.03	0.8649	0.32	0.0877	-0.22	0.2482	-0.01	0.9756	0.01	0.9756
Ergometrinine	-0.18	0.3400	-0.23	0.2341	0.26	0.1760	0.39	0.0344	0.31	0.1004	0.38	0.0397	0.54	0.0027	-0.36	0.0560	-0.02	0.9163	0.02	0.9163
Ergot alkaloids	-0.18	0.3373	-0.23	0.2341	0.27	0.1601	0.40	0.0329	0.31	0.1004	0.38	0.0406	0.53	0.0031	-0.35	0.0621	-0.02	0.9082	0.02	0.9082
Beauvericin	0.12	0.5199	0.15	0.4453	-0.06	0.7651	-0.05	0.8140	0.06	0.7602	0.03	0.8931	0.16	0.4096	-0.24	0.2038	-0.04	0.8520	0.04	0.8520
Bikaverin	-0.22	0.2597	0.08	0.6713	0.13	0.5098	0.47	0.0105	0.47	0.0103	0.49	0.0069	0.31	0.0997	-0.58	0.0011	-0.09	0.6563	0.09	0.6563
Epiequisetin	-0.15	0.4369	-0.24	0.2124	0.25	0.1847	0.04	0.8374	0.08	0.6620	0.02	0.9205	0.02	0.9266	-0.23	0.2343	0.07	0.7347	-0.07	0.7347
Equisetin	-0.12	0.5488	-0.20	0.2883	0.18	0.3574	0.04	0.8175	0.11	0.5543	0.01	0.9592	-0.06	0.7620	-0.20	0.3027	0.06	0.7453	-0.06	0.7453
Fumonisin B1	-0.27	0.1580	-0.16	0.4030	0.22	0.2415	0.54	0.0028	0.54	0.0027	0.52	0.0041	0.36	0.0516	-0.56	0.0014	-0.06	0.7618	0.06	0.7618
Fumonisin B2	-0.21	0.2699	-0.09	0.6593	0.21	0.2690	0.55	0.0018	0.56	0.0016	0.57	0.0013	0.32	0.0917	-0.47	0.0093	-0.10	0.6045	0.10	0.6045
Fumonisin B3	-0.11	0.5739	0.02	0.9198	0.18	0.3527	0.47	0.0098	0.42	0.0233	0.35	0.0651	0.27	0.1640	-0.50	0.0057	-0.16	0.4118	0.16	0.4118
Moniliformin	-0.07	0.7240	0.16	0.4028	-0.36	0.0518	0.14	0.4770	0.13	0.5010	0.17	0.3778	0.36	0.0573	-0.56	0.0017	-0.08	0.6773	0.08	0.6773
Monocerin	-0.03	0.8938	0.05	0.8002	-0.20	0.2933	0.06	0.7629	0.26	0.1745	0.10	0.6030	-0.22	0.2430	-0.14	0.4531	0.26	0.1651	-0.26	0.1651
Nivalenol	-0.10	0.5933	-0.11	0.5731	0.07	0.7238	0.31	0.1061	0.37	0.0501	0.29	0.1315	0.08	0.6679	-0.01	0.9708	0.18	0.3393	-0.18	0.3393
Siccanol	-0.10	0.5941	-0.03	0.8952	-0.01	0.9763	-0.22	0.2602	-0.14	0.4593	-0.19	0.3202	-0.31	0.1020	0.14	0.4745	0.22	0.2564	-0.22	0.2564
Zearalenone	-0.28	0.1366	-0.10	0.6087	0.09	0.6269	0.25	0.1822	0.26	0.1731	0.23	0.2379	-0.04	0.8517	-0.18	0.3466	0.32	0.0945	-0.32	0.0945
from *Fusarium*	-0.21	0.2856	-0.15	0.4445	0.12	0.5258	0.01	0.9644	0.09	0.6518	0.04	0.8558	-0.16	0.3943	0.06	0.7476	0.25	0.1906	-0.25	0.1906
Ascochlorin	0.10	0.5897	-0.06	0.7725	0.01	0.9472	0.05	0.7938	-0.05	0.8014	-0.23	0.2294	-0.46	0.0120	0.28	0.1420	0.18	0.3416	-0.18	0.3416
Barceloneic acid	0.20	0.2942	-0.22	0.2459	0.02	0.9104	-0.03	0.8619	0.12	0.5295	0.03	0.8791	-0.12	0.5332	0.09	0.6409	0.08	0.6931	-0.08	0.6931
Cytochalasin D	0.10	0.6156	-0.09	0.6459	-0.15	0.4514	-0.05	0.8068	0.00	0.9857	-0.10	0.5910	0.04	0.8381	0.01	0.9572	0.03	0.8785	-0.03	0.8785
Destruxin B	-0.15	0.4437	-0.20	0.2955	-0.08	0.6629	-0.45	0.0154	-0.37	0.0480	-0.14	0.4717	0.07	0.7034	0.15	0.4306	0.08	0.6672	-0.08	0.6672
Ilicicolin B	-0.05	0.8149	0.16	0.4187	-0.21	0.2684	0.11	0.5861	0.20	0.2886	0.01	0.9480	-0.25	0.1962	0.13	0.4857	0.34	0.0717	-0.34	0.0717
Neoechinulin A	0.15	0.4226	0.38	0.0419	-0.26	0.1791	-0.12	0.5495	-0.03	0.8823	-0.06	0.7479	0.03	0.8859	-0.40	0.0298	-0.14	0.4707	0.14	0.4707
from other fungi	-0.04	0.8507	0.12	0.5300	0.19	0.3271	0.19	0.3162	0.14	0.4849	0.15	0.4303	0.27	0.1585	0.06	0.7457	-0.15	0.4296	0.15	0.4296
Cycloaspeptide A	-0.11	0.5660	-0.01	0.9716	0.11	0.5693	-0.16	0.4044	0.03	0.8874	0.02	0.9242	-0.13	0.5115	0.09	0.6278	0.12	0.5384	-0.12	0.5384
Flavoglaucin	0.10	0.5915	0.04	0.8223	-0.11	0.5592	0.10	0.6075	0.15	0.4338	0.03	0.8679	0.06	0.7529	-0.46	0.0115	-0.05	0.8118	0.05	0.8118
Griseofulvin	0.01	0.9667	-0.02	0.9235	-0.13	0.4966	-0.10	0.6010	-0.02	0.9086	-0.20	0.3067	-0.35	0.0618	0.35	0.0631	0.34	0.0721	-0.34	0.0721
Oxaline	0.18	0.3482	-0.21	0.2835	0.02	0.9105	-0.09	0.6551	-0.03	0.8921	-0.24	0.2019	-0.21	0.2849	0.27	0.1570	0.13	0.5061	-0.13	0.5061
Phenopyrrozin	0.05	0.7982	-0.19	0.3179	0.30	0.1108	0.06	0.7683	-0.03	0.8934	0.02	0.9246	0.08	0.6751	0.15	0.4461	0.00	0.9878	0.00	0.9878
Questiomycin derivate	0.01	0.9650	-0.06	0.7601	0.41	0.0282	-0.29	0.1296	-0.23	0.2348	-0.19	0.3174	-0.12	0.5185	-0.12	0.5482	-0.26	0.1733	0.26	0.1733

From *Penicillium*	-0.08	0.6650	0.23	0.2399	0.10	0.5993	-0.19	0.3290	-0.18	0.3497	-0.11	0.5768	0.32	0.0863	-0.05	0.7965	-0.30	0.1134	0.30	0.1134
Total fungal metabolites	0.28	0.1483	0.23	0.2200	0.03	0.8670	0.03	0.8955	-0.04	0.8492	-0.06	0.7586	0.21	0.2831	-0.12	0.5304	-0.35	0.0603	0.35	0.0603
